# Sequencing of the chloroplast genome of Taimo, a paddy cultivar of Taro (*Colocasia esculenta* (L.) Schott) in the Ryukyu Archipelago

**DOI:** 10.1080/23802359.2025.2505789

**Published:** 2025-05-20

**Authors:** Reina Takaesu, Yukihiro Kinjo, Crystal-Leigh Clitheroe, Haruki Sunagawa, Thomas Bourguignon, Seikoh Saitoh

**Affiliations:** a Department of Regional Economics and Environmental Policy, Okinawa International University, Ginowan, Japan; b Graduate School of Environment and Information Sciences, Yokohama National University, Hodogaya, Yokohama, Japan; c Okinawa Institute of Science and Technology Graduate University, Onna-son, Japan; d Okinawa Churashima Research Institute, Okinawa Churashima Foundation, Motobu, Japan

**Keywords:** Aroids, Okinawa, dispersal, traditional crop

## Abstract

Taimo is a paddy cultivar of Taro (*Colocasia esculenta* (L.) Schott) that is traditionally consumed in the Ryukyu Archipelago, Japan. Its origin remains unknown, although it has been identified as belonging to a haplotype commonly found in tropical and subtropical regions. However, previously developed genetic markers were insufficient to resolve the genetic relationships among cultivars that share the same haplotype. To address this limitation, we sequenced the complete chloroplast genome of cv. Taimo. The comparison of the obtained sequence with the currently published complete and partial chloroplast genomes revealed that cv. Taimo is most closely related to the paddy cultivar Lipu from southern mainland China. These findings suggest that the ancestors of cv. Taimo were introduced to the Ryukyus from China.

## Introduction

Taimo is a cultivar of Taro (*Colocasia esculenta* (L.) Schott, 1832) cultivated in the Ryukyu Archipelago, the southernmost part of Japan. Unlike most Taro cultivars in Japan, which are grown in dry fields, Taimo is cultivated in paddy fields. The name “Ta-imo” means “tuber of the paddy field.” Historical literature suggests that Taro cultivation in the Ryukyus dates back to the fifteenth century and that Taro may have been an essential food source before the introduction of sweet potato in the seventeenth century (Ankei 1985; Sasaki 2003). Moreover, Taimo has been used as an ingredient in traditional festive dishes consumed during annual events related to indigenous faith in local communities (Shimono 1980).

Taro domestication has occurred multiple times independently within its naturally distributed areas, with its cultivars being subsequently introduced to other regions (Chaïr et al. 2016; Ahmed et al. 2020). Taimo may have been introduced to the Ryukyus from neighboring areas, such as mainland China, Taiwan, or the Philippines, rather than being originally domesticated in the Ryukyus. However, the wild-type Taro (*C. esculenta* var. *aquatilis*) is naturally distributed in this area (Ankei 1985, 1987; Matthews 1991, 1995).

**Figure 1.**
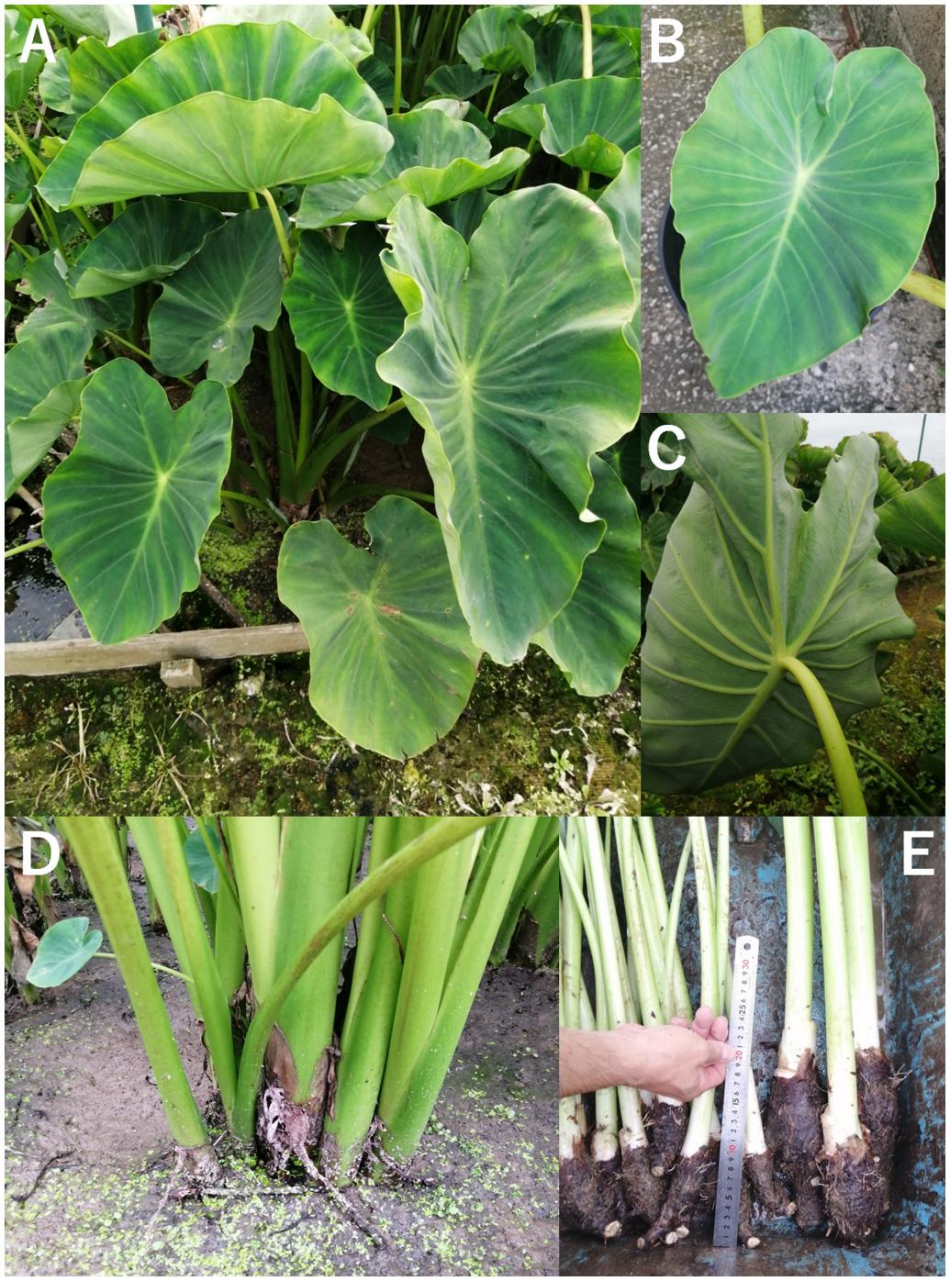
*Colocasia esculenta* cultivar taimo cultivated in a paddy field in the Ryukyu Archipelago (Okinawa, Japan). (A–C) leaf blades are ovate, undulate, and have a single sinus between the two posterior lobes, and the costae, veins, and lamina are green. Each leaf blade has three main costae: one anterior and two posterior, with 8–10 primary lateral veins branching from the anterior costa and either of the two posterior costae. (A, D, E) Mother, side shoots, and corms. Side corms branch extensively from the mother corm under cultivated conditions, while stolon formation or flowering was not observed (Ishikawa, T., personal communication). (C–E) Petioles are green with a white basal part. (E) Corms harvested by the farmer; mother and side corms are positioned on the right and left of the ruler (30 cm in length), respectively. The roots that emerge from the corm, as well as the skin and flesh (cortical parenchyma) of the corm, are white (not shown). Anthocyanin pigmentation was not observed on any part, except for subtle pigmentation that was occasionally found at the joint between the leaf blade and petiole (not shown). Photos were taken by S. Saitoh: at Oyama, Ginowan on September 11, 2024 (E) and February 11, 2025 (A, C–D); a plant grown from a cut shoot in a pot in the laboratory on February 15, 2025 (B).

Taimo is diploid (*n* = 28) and does not produce stolons or flowers or may rarely produce flowers (Ankei 1987). Farmers propagate this cultivar vegetatively by using cuttings from shoots. The parent and side corms are utilized as food sources. Hotta (1970) classified the cultivar group *Senkuchi*, which characteristically produces a large mother corm as well as numerous smaller side corms that bear leaves, under *Colocasia esculenta* var. *esculenta* and included this cultivar within the group. Previous studies have shown variation in Taimo’s morphology and color between islands (Ankei 1987, 1993, 1995). The widely farmed cultivar is known as the “white corm type,” characterized by a lack of or only subtle anthocyanin pigmentation in the corm’s skin, leaf edges, veins, and petioles (Ankei 1987; Figure 1).

Ahmed et al. (2020) investigated the phylogeny of Taro, including cultivars and wild types in the Asia-Pacific region, based on genetic markers from chloroplast DNA. They identified three distinct clades: the first clade (CI), mainly distributed in tropical regions; the second clade (CII), found in temperate regions; and the third clade (CIII), whose haplotypes were found only in wild populations. CI and CII included both cultivars and wild types. The data indicated that Taimo (accession ID: CESJP10) belongs to the haplotype Type 1 in the CI clade (CI.T1), which was the most frequently observed haplotype in their study (Ahmed et al. 2020). This haplotype was found across broad tropical regions, including Southeast Asia and Oceania, as well as in the Japanese mainland. Although the morphology, particularly the shape and pigmentation of leaves and corms, varied considerably among the CI.T1 accessions, their DNA sequences at the six genetic marker loci analyzed were similar, making it challenging to discern genetic relationships among them.

Therefore, we sequenced the chloroplast genome of Taimo and compared it with the published complete and partial Taro chloroplast genomes to examine genetic variations within and between the haplotypes. Based on these comparisons, we determined the potential region from which Taimo was introduced to the Ryukyus.

## Materials and methods

Taimo (white corm type) samples were collected from a paddy field on Okinawa Island, the main island of the Ryukyus (26.285°N, 127.748°E). A voucher specimen of this cultivar was deposited in the Okinawa Churashima Research Institute (https://churashima.okinawa/en/ocrc/, Dr. H. Sunagawa: h-sunagawa@okichura.jp) under accession no. CSC24091101.

DNA was extracted from the leaf tissue using the DNeasy Plant Mini Kit (Qiagen). A DNA library for shotgun sequencing on the DNBSEQ platform (MGI Tech, Shenzhen, China) was prepared following the manufacturer’s protocol and sequenced using the DNBSEQ-T7 sequencer at the Bioengineering Lab Co., Ltd. (Sagamihara, Japan).

The chloroplast genome was assembled using SPAdes assembler (v3.15.01, Prjibelski et al. 2020). The resulting contigs corresponding to the large single-copy (LSC), small single-copy (SSC), and inverted repeat (IR) regions were aligned to the reference genome using GSAlign software (v1.0.22; Lin and Hsu 2020). Boundaries between the LSC, SSC, and the two IR region copies were sequenced by Sanger sequencing to confirm those assembled from short reads. The sequencing depth was evaluated by mapping reads onto the assembled genome using Bowtie 2 software (v2.3.5.1, Langmead and Salzberg 2012) and SAMtools software (Li et al. 2009).

Annotation of the chloroplast genomes was initially performed using the CPGAVAS2 annotation pipeline (Shi et al. 2019). The annotated sequences were then manually edited by comparing them with published genomes from the same species (JN105689.1 and JN105690.1 from Ahmed et al. 2012; MT447084.1 and MT447085.1 from Jia et al. 2023; OP589403.1 from Yin et al. 2023). The annotated genome was visualized using OGDRAW (Greiner et al. 2019).

Phylogenetic inference was performed based on the complete chloroplast genomes of cv. Taimo and the five genomes of the other Taro cultivars mentioned above. *Steudnera colocasiifolia* (MT161479.1; Abdullah et al. 2021) was used as an outgroup. The chloroplast genomes, including the LSC, SSC, and only one of the two IR copies (to prevent overrepresentation of the IR regions), were aligned using MAFFT (v7.520; Katoh and Standley 2013). Poorly aligned regions were removed from the multiple sequence alignment using trimAl (v1.2; Capella-Gutiérrez et al. 2009), which resulted in a final sequence alignment of 138,365 nucleotides. Maximum Likelihood inference of the phylogenetic tree was performed using the online version of IQ-TREE (Nguyen et al. 2015; Trifinopoulos et al. 2016) with default settings, where the substitution model TVM + F + I was automatically selected. In addition, we performed phylogenetic analysis using genetic markers following the method described by Ahmed et al. (2020) and utilizing the publicly available data deposited by them. This analysis aimed to confirm the haplotype of the sequenced genome in this study (CI.T1) and identify cultivars that share the same haplotype.

Single nucleotide variations (SNVs) and insertions/deletions (indels) were searched in the abovementioned complete Taro chloroplast genomes and partial genomes of haplotype CI.T1 published by Ahmed et al. (2013, 2020). The genomes were aligned against the genome of Taimo using GSAlign software (Lin and Hsu 2020).

## Results

**Figure 2.**
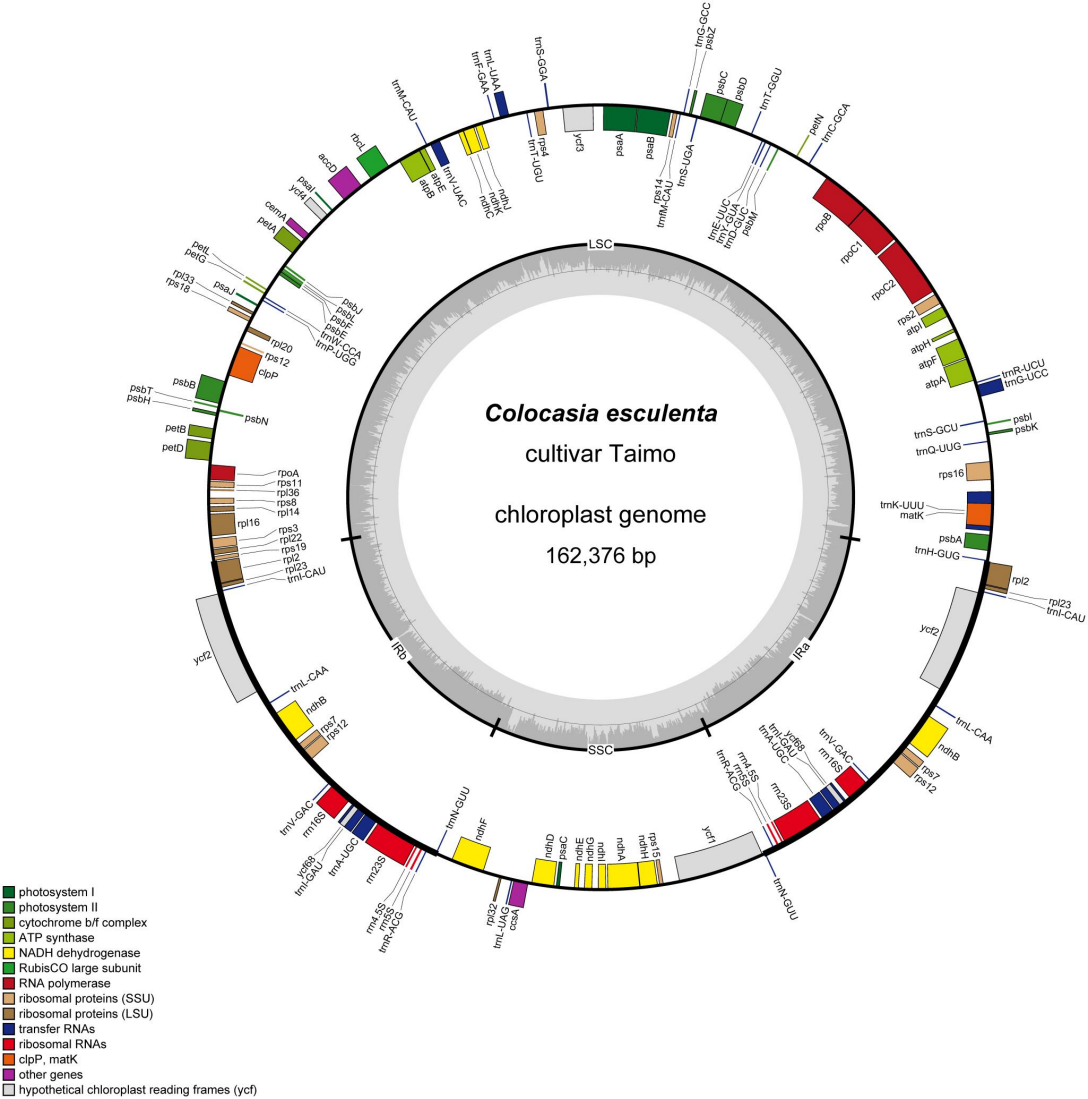
Genome map of Taimo chloroplast, a Taro paddy cultivar from the Ryukyu Archipelago. The genome was visualized using OGDRAW (Greiner et al. 2019). The inner track displays the GC content and the positions of the LSC, SSC, IRa, and IRb regions. The outer circle corresponds to the genes: colored boxes on the inner side of the circle represent genes transcribed clockwise, whereas those on the outer side are transcribed counterclockwise.

The sequenced plastid genome of Taimo was 162,376 bp long and included an 89,642 bp LSC region, a 22,188 bp SSC region, and two copies of a 25,273-bp IR region (Figure 2). The median sequencing depth of the chloroplast genome was estimated at 1,126× (Figure S1). The genome annotation revealed 131 genes, comprising 86 protein-coding genes, 8 rRNA genes, and 37 tRNA genes. The structures of spliced genes are shown in Figure S2.

**Figure 3.**
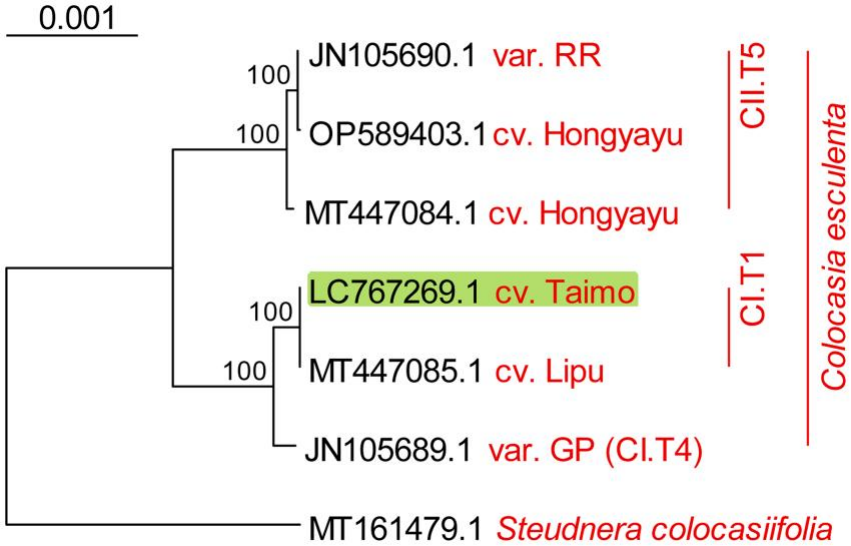
Phylogenetic tree of taro accessions was inferred using the maximum likelihood based on the complete chloroplast genome. Taimo, the *Colocasia esculenta* variety sequenced in this study, is highlighted in green. The complete chloroplast genomes include var. GP (CESNZ03, GenBank acc. no. JN105689.1) and RR (CESNZ02, JN105690.1) from Ahmed et al. (2012); cv. Hongyayu (redbud) (MT447084.1) and Lipu (MT447085.1) from Jia et al. (2023); cv. Hongyayu (OP589403.1, Yin et al. 2023); and cv. Taimo (LC767269.1, present study). *Steudnera colocasiifolia* (MT161479.1) from Abdullah et al. (2021) was used as an outgroup. Haplotypes based on the six loci from the chloroplast genomes were determined for the *Colocasia* accessions through phylogenetic analysis with data from Ahmed et al. (2020) (see Figure S3). Codes in the figure, such as Cx.Tx, indicates the haplotypes based on the six loci from the chloroplast genome (see text).

The phylogenetic tree based on the complete chloroplast genomes (Figure 3) indicated that the Chinese cultivar Lipu (MT447085.1) was closest to Taimo. The phylogenetic tree based on the six chloroplast loci (Figure S3) confirmed that cv. Taimo and cv. Lipu belonged to haplotype CI.T1, as both were placed in the same position as other accessions of haplotype CI.T1—namely, the Japanese cultivar Tono-imo (CESJP01 in Figure S3) and an ornamental sample from New Zealand (CESNZ14, var. *fontanesii*), whose genomes, albeit partial, were available.

Examination of genomic variations in the published partial or complete Taro chloroplast genomes compared with cv. Taimo (Table S1) revealed that the NZ accession CESNZ14 (partial genome, 17,086 bp) differed by seven SNVs and six indels. The Japanese cultivar Tono-imo CESJP01 (partial genome, 14,991 bp) exhibited one SNV and two indels, whereas the complete plastid genome of the Chinese cultivar Lipu (MT447085.1) displayed no SNVs and contained a 77-bp insertion. The number of observed variations in the chloroplast genomes compared with cv. Taimo has been categorized and summarized in Table S1.

## Discussion

The chloroplast genome of the paddy cultivar Taimo harbored an equivalent number and order of genes compared with other published Taro chloroplast genomes (Ahmed et al. 2012; Jia et al. 2023; Yin et al. 2023).

The phylogenetic inference confirmed that Taimo belongs to haplotype CI.T1 among the six loci-based haplotypes, as indicated by Ahmed et al. (2020). Further examination of available partial and complete Taro chloroplast genomes within CI.T1 indicated that the Chinese cultivar Lipu was the closest to Taimo among the currently sequenced Taro cultivars; no SNV was detected between the complete chloroplast genomes of Taimo and Lipu. In contrast, more SNVs were observed in the other two accessions with partial genomes. This finding suggests that they shared a common ancestral cultivar or that their ancestral wild types were genetically close despite being domesticated independently.

The Chinese cultivar Lipu is cultivated in Lipu, Guangxi (a southern region of mainland China bordering Vietnam). This cultivar shares several common features with Taimo: they are suited for cultivation in paddy fields, and their corms are sticky and fragrant (Ankei 1985; Kinjo et al. 2003; Jia et al. 2023).

Based on the historical literature compiled in the early eighteenth century, Lipu was originally introduced to Guangxi from Fujian, an eastern coastal province of China (Jiang and Ou 2016). Archaeological evidence indicates that direct trade between the coastal areas of mainland China and the Ryukyus began in the late twelfth century (Pearson 2007). Another historical source reported the cultivation of paddy Taro in Okinawa in the sixteenth century (Sasaki 2003). The historical and archaeological evidence presented by these studies leads to the hypothesis that Taimo was introduced from Fujian or nearby areas to the Ryukyus during the Medieval period.

A more detailed history of their introduction to the currently cultivated areas could be elucidated by future sequencing of the complete chloroplast genomes of related cultivars as well as population genetics studies using microsatellite markers or SNVs from their nuclear genomes.

## Conclusions

Sequencing the complete chloroplast genome of cv. Taimo provided insights into its spread. Additionally, this sequence is a valuable diagnostic resource for distinguishing between other cultivars of the same haplotype. Future studies integrating nuclear genome sequencing and a broader sampling of cultivars across different regions will further refine our understanding of the genetic history and domestication pathways of Taro.

## Supplementary Material

supplementary_materials_20250401.docx

## Data Availability

The genome sequence data that support the findings of this study are openly available in GenBank of NCBI at https://www.ncbi.nlm.nih.gov under the accession no. LC767269. The associated BioProject, SRA, and Bio-Sample numbers are PRJDB15812, DRX595706, and SAMD00602903 respectively.
