# Supplementary material for: Sequencing of the chloroplast genome of Taimo, a paddy cultivar of Taro (Colocasia esculenta (L.) Schott) in the Ryukyu Archipelago
Source: Mitochondrial DNA B Resour. 2025 May 20;10(6):508–12. doi: 10.1080/23802359.2025.2505789 (PMC12093797; doi:10.1080/23802359.2025.2505789)
Supplement: supplementary_materials_20250401.docx [file TMDN_A_2505789_SM4928.docx]

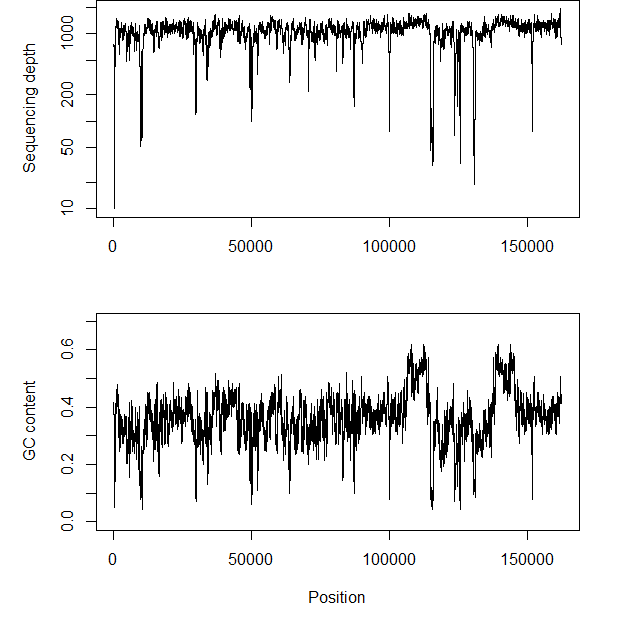


**Figure S1.** Sequencing depth (upper panel) and GC content (lower panel) of the chloroplast genome of cv. Taimo. High-throughput sequencing generated 27.2 million paired-end reads (2 × 150 bp), totaling 8.15 Gbp of data, with an evaluated mean insert size of 250 bp. The sequencing depth of the chloroplast genome ranged from 10 to 1,959, with an average of 1,086. AT-rich regions exhibited lower sequencing depth, and GC content was positively correlated with sequencing depth (R = 0.79). Overall, 2.09% of the total data were derived from the plastid genome based on read-mapping depth.


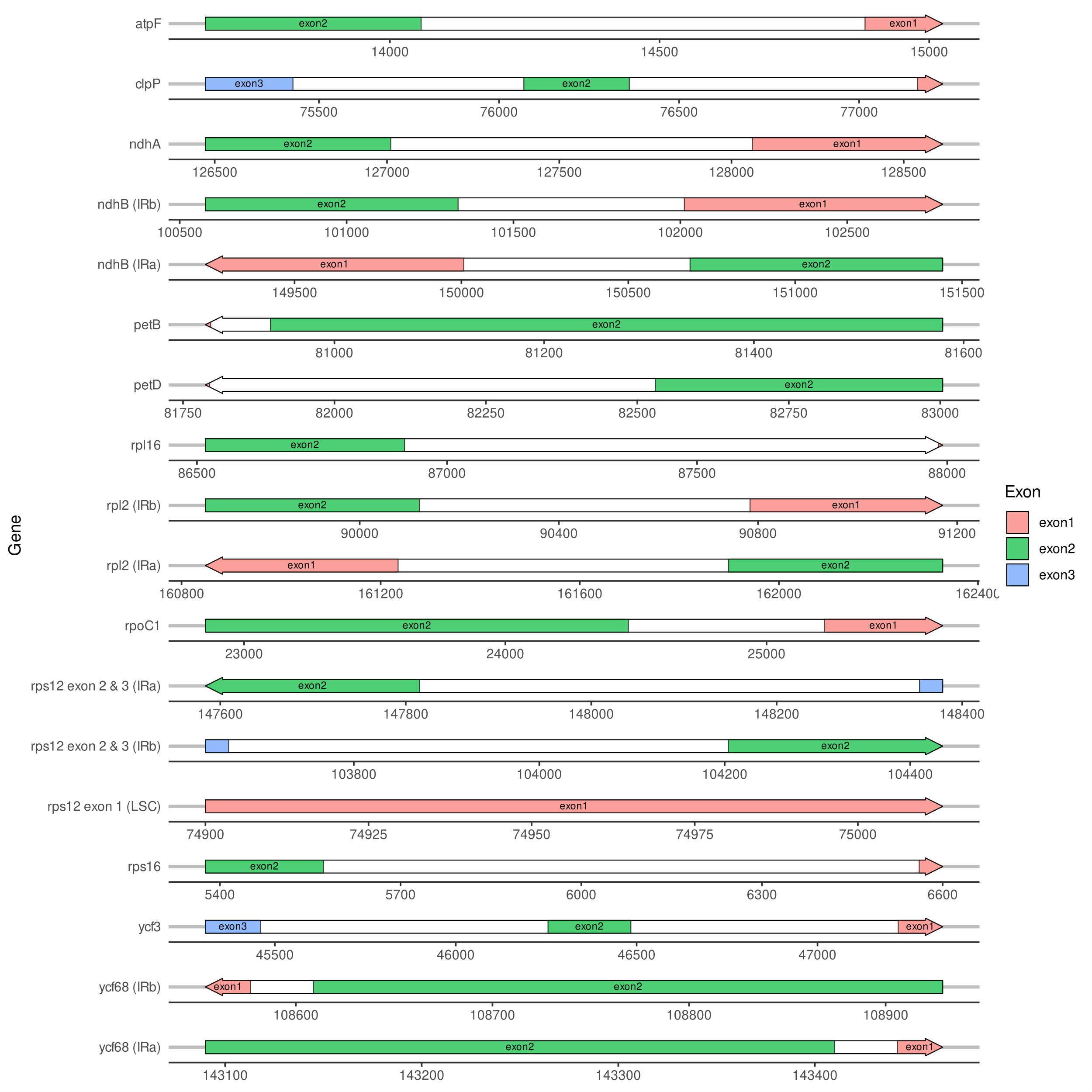


**Figure S2.** Map of spliced genes in the chloroplast genome of cv. Taimo. Exons and introns are represented by the filled and empty segments of the arrows, respectively. The orientation of each arrow indicates the transcriptional direction. The ribosomal protein S12 gene (*rps*12) is trans-spliced, with exon 1 transcribed from the LSC region and exons 2 and 3 transcribed from the IRa or IRb region.

(A) (B)

**Figure S3.** Phylogenetic tree of Taro accessions inferred using Maximum Likelihood based on six loci from the chloroplast genome. (A) Phylogram; (B) Cladogram (displaying only tree topology, without branch length information). Thirty-six *Colocasia* accessions, whose sequences from all six loci were available from NCBI GenBank, as indicated in Ahmed et al. (2020), were analyzed alongside those from Taimo and three other complete chloroplast genomes. *Steudnera* sp. SSPVN01 was used as an outgroup. Taimo, the *Colocasia esculenta* variety sequenced in this study, is highlighted in green. The topology of the resultant tree was identical to that in Ahmed et al. (2020). Clades I–III were numbered following Ahmed et al. (2020). GenBank accession numbers are listed in Table S1 of Ahmed et al. (2020). Codes in parentheses in the figure, such as Cx.Tx, indicate haplotypes based on six loci from the chloroplast genome, as assigned by Ahmed et al. (2020). Daggers (†) indicate accessions that Ahmed et al. (2020) morphologically identified as species other than *C*. *esculenta*. Asterisks (*) indicate accessions with complete chloroplast genomes available. Complete chloroplast genomes include var. GP (CESNZ03, GenBank acc. no. JN105689.1) and RR (CESNZ02, JN105690.1) from Ahmed et al. (2012); cv. Hongyayu (redbud) (MT447084.1) and Lipu (MT447085.1) from Jia et al. (2023); cv. Hongyayu (OP589403.1, Yin et al., 2023); and cv. Taimo (LC767269.1, present study). **Analytic procedure:** The six-loci sequences were aligned per gene using MUSCLE (Edgar, 2004) and then concatenated. Poorly aligned regions were removed from the multiple sequence alignment using trimAl (v1.2; Capella-Gutiérrez et al., 2009), which resulted in a final sequence alignment of 2,426 nucleotides. Maximum Likelihood inference of the phylogenetic tree was performed using PhyML (v3.0; Guindon et al., 2010) with the Generalized Time Reversible model and the Gamma-distributed rates among sites and Invariant sites (GTR+G+I) model. Heuristic tree topology searching was performed using the Subtree-Pruning-Regrafting method.

**Table S1.** Genomic variations between chloroplast genomes of cv. Taimo and other Taro cultivars.

^1^ Accession numbers as listed in Ahmed et al. (2020) or NCBI GenBank.

^2^ Chloroplast haplotypes as determined in Ahmed et al. (2020). For MT447084.1, MT447085.1, and OP589403.1, haplotypes were assigned based on sequence alignment with data from Ahmed et al. (2020).

^3^ Information source: Data for CESJP01, CESNZ01, CESNZ02, CESNZ03, and CESNZ14 are from Table S1 in Ahmed et al. (2020); data for MT447084.1 and MT447085.1 are from Jia et al. (2023); data for OP589403.1 are from Yin et al. (2023).

^4^ GenBank accession numbers for CESJP01, CESNZ01, CESNZ02, CESNZ03, and CESNZ14 are provided in Ahmed et al. (2013).
